# Replication of Hepatitis E Virus (HEV) in Primary Human-Derived Monocytes and Macrophages In Vitro

**DOI:** 10.3390/vaccines8020239

**Published:** 2020-05-21

**Authors:** Ibrahim M. Sayed, Mohamed Ismail Seddik, Marwa A. Gaber, Saber H. Saber, Sahar A. Mandour, Mohamed A. El-Mokhtar

**Affiliations:** 1 Department of Medical Microbiology and Immunology, Faculty of Medicine, Assiut University, Assiut 71515, Egypt; ibrahim.ibrahim@aun.edu.eg; 2 Department of Pathology, School of Medicine, University of California, San Diego, CA 92093, USA; 3 Department of Clinical Pathology, Faculty of Medicine, Assiut University, Assiut 71515, Egypt; moh.ismail310@aun.edu.eg; 4 Department of Medical Biochemistry, Faculty of Medicine, Assiut University, Assiut 71515, Egypt; marwagaber@aun.edu.eg; 5 Laboratory of Molecular Cell Biology, Department of Zoology, Faculty of Science, Assiut University, Assiut 71515, Egypt; Saberhassan@aun.edu.eg; 6 Department of Microbiology and Immunology, Faculty of Pharmacy, Deraya University, Minia 11566, Egypt; sahar.mandour@deraya.edu.eg

**Keywords:** HEV infection, PBMCs, bone marrow, ds-RNA, capsid protein, immune response, chronic, extrahepatic, hematological disorders

## Abstract

HEV is the most causative agent of acute viral hepatitis globally. HEV causes acute, chronic, and extrahepatic manifestations. Chronic HEV infection develops in immunocompromised patients such as organ transplant patients, HIV-infected patients, and leukemic patients. The source of chronic HEV infection is not known. Also, the source of extrahepatic manifestations associated with HEV infection is still unclear. Hepatotropic viruses such as HCV and HBV replicate in peripheral blood mononuclear cells (PBMCs) and these cells become a source of chronic reactivation of the infections in allograft organ transplant patients. Herein, we reported that PBMCs and bone marrow-derived macrophages (BMDMs), isolated from healthy donors (n = 3), are susceptible to HEV in vitro. Human monocytes (HMOs), human macrophages (HMACs), and human BMDMs were challenged with HEV-1 and HEV-3 viruses. HEV RNA was measured by qPCR, the marker of the intermediate replicative form (ds-RNA) was assessed by immunofluorescence, and HEV capsid protein was assessed by flow cytometry and ELISA. HEV infection was successfully established in primary HMOs, HMACs, and human BMDMs, but not in the corresponding cells of murine origin. Intermediate replicative form (ds RNA) was detected in HMOs and HMACs challenged with HEV. The HEV load was increased over time, and the HEV capsid protein was detected intracellularly in the HEV-infected cells and accumulated extracellularly over time, confirming that HEV completes the life cycle inside these cells. The HEV particles produced from the infected BMDMs were infectious to naive HMOs in vitro. The HEV viral load was comparable in HEV-1- and HEV-3-infected cells, but HEV-1 induced more inflammatory responses. In conclusion, HMOs, HMACs, and human BMDMs are permissive to HEV infection and these cells could be the source of chronic and recurrent infection, especially in immunocompromised patients. Replication of HEV in human BMDMs could be related to hematological disorders associated with extrahepatic manifestations.

## 1. Introduction

HEV infection is endemic in developing and developed countries [1,2]. HEV is the most causative agent of acute viral hepatitis globally that infects about 20 million annually [3]. HEV belongs to the *Orthohepevirus* genus of the *Hepeviridae* family, and there are eight known HEV genotypes [2]. HEV-1 and HEV-2 are associated with waterborne infection in developing countries and they infect humans only [3]. HEV-3 and HEV-4 are common in developed countries and they are zoonotic. Pigs, wild boars, deer, and rabbits are the main reservoirs for these isolates, and the infection is mainly transmitted by the ingestion of contaminated animal products [4,5,6]. Infection with those two genotypes has also been documented by blood transfusion [7]. HEV-5 and HEV-6 infect wild boars in Japan, and HEV-8 infects Bactrian camels; they are not confirmed as human pathogens [8,9]. HEV-7 infect camels in the Middle East [10]; an infection was documented in a liver transplant patient who regularly consumed camel meat and milk [11].

HEV is a small icosahedral positive-sense single-strand RNA virus that includes three open reading frames (ORF). ORF1, located at the 5′ end of the genome, represents two-thirds of the viral genome and encodes nonstructural enzymes that are responsible for viral replication such as RNA-dependent RNA polymerase, helicase, and cysteine protease. ORF2, located at the 3′ end of the genome, encodes a structural capsid protein, and ORF3, which overlaps with ORF1 and ORF2, encodes a small phosphoprotein that plays a role in viral morphogenesis [12].

HEV infection was first recognized as an acute self-limiting disease, but these infections can evolve to chronicity in immunocompromised patients such as organ transplant patients, HIV-infected patients, and patients with hematological disorders [13,14,15]. Extrahepatic manifestations have been reported in association with HEV infection such as neurological disorders, glomerulonephritis, cryoglobulinemia, acute pancreatitis, thrombocytopenia, and hemolytic anemia [16,17]. Not all of the extrahepatic targets for HEV have been identified. Recently, we reported that non-decidualized primary human endometrial stromal cells (PHESCs), precursors for the decidua and placenta, are susceptible to HEV infection [18]. 

Several studies have reported the replication of hepatotropic viruses such as HAV, HBV, and HCV in peripheral blood mononuclear cells (PBMCs). PBMCs could serve as a reservoir for these hepatotropic viruses and sources for chronic infection. [19,20,21]. In addition, the replication of hepatotropic viruses in the myeloid cells and bone marrow could be linked to the hematological disorders associated with the infection with these viruses [22,23]. Hematological manifestations are associated with HEV infection such as hepatitis-associated aplastic anemia, secondary hemophagocytic syndrome, severe thrombocytopenia, and hemolytic anemia [24]. However, the data available on the replication of HEV in PBMCs are limited. To our knowledge, there is only one study that reported the presence of positive (+)-strand, but not negative (−)-strand HEV RNA in the PBMCs of acute HEV patients [25]. However, the methodology used in this study to detect (−)-strand HEV RNA was not highly sensitive [25]. Moreover, no data are available on the replication of HEV in PBMCs of chronic HEV patients or patients with extrahepatic manifestations.

Herein, we investigated the replication of HEV in human-derived monocytes (HMOs) and/or macrophages (HMACs) and human bone marrow-derived macrophages (BMDMs), isolated from healthy donors, in vitro. To our knowledge, our study is the first report that shows the replication of HEV in HMOs, HMACs, and human BMDMs

## 2. Materials and Methods

### 2.1. Subjects

Bone marrow aspirates were obtained from the end of femurs removed from patients (n = 3) undergoing orthopedic hip reconstruction operations. Blood samples were collected from the same donors. All donors were screened accroding to the protocol of Assiut University Hospitals and they tested negative for viral hepatitis markers (HAV, HBV, HCV, and HEV markers) at the time of sample collection as described previously [18,26,27]. All participating subjects provided written informed consent, and the protocol of infection of peripheral blood mononuclear cells (PBMCs) with HEV was approved by the Institutional Review Board (IRB) at the Faculty of Medicine, Assiut University, Egypt, in accordance with the provisions of the Declaration of Helsinki (IRB no 17200190).

### 2.2. Animals

Balb/c (n = 3) male mice (8–12 weeks) were obtained from Animal Facility, Faculty of Medicine, Assiut University, Egypt. Blood and bone marrow were collected from the mice after euthanasia. All experiments were done and approved in accordance with by the Institutional Review Board (IRB) at the Faculty of Medicine, Assiut University, Egypt. (IRB no 17200190)

### 2.3. Cell Lines

Immortalized human monocytes cells (THP-1) and mouse macrophage cells (RAW 264.7) were kindly provided by the VACSERA-Cell Culture Unit, Cairo. THP-1 were maintained in Roswell Park Memorial Institute (RPMI-1640) medium (Gibco, ThermoFisher Scientific, Grand Island, NY, USA) containing 10% fetal bovine serum (FBS) (Gibco, ThermoFisher Scientific) and kept at 37 °C and 5% CO_2_. RAW 264.7 were maintained in Dulbecco’s modified Eagle’s media (DMEM) (Gibco, ThermoFisher Scientific, Grand Island, NY, USA) under the same condition.

### 2.4. HEV Inoculums

HEV inoculums were isolated from the stool of acute HEV-infected patients admitted for diagnostic purposes to Assiut University hospitals as described previously [18].

### 2.5. Isolation of Mouse Monocytes and Bone Marrow and Differentiation into Macrophages In Vitro

Mouse monocytes were isolated from whole blood by immunomagnetic negative selection using the EasySep™ Mouse Monocyte Isolation Kit (Stem Cell, Technologies, Cambridge, MA, USA) according to the manufacturer’s instructions. The purity of the isolated cells was analyzed by flow cytometry after staining with PE anti-mouse CD14 (BioLegend, CA, USA) and FITC anti-mouse CD11b antibodies (BioLegend, USA).

For isolation of cells from the bone marrow, the femurs were removed and stripped of muscle, exposed bone was washed in 70% EtOH, and a small piece at both extremities of the femur head was cut using scissors. Bone marrow cells were flushed with cold phosphate buffered saline (PBS, and the cell suspension was dissolved in RBCs lysis buffer (1X, Thermofischer, Grand Island, NY, USA). Then, the cell suspension was filtered through a cell strainer (70 μm) and centrifuged. To differentiate blood monocytes and bone marrow-derived monocytes into macrophages, the cell pellets were dissolved in RPMI medium containing 1% penicillin/streptomycin and macrophage-colony stimulating factor (GM-CSF, conc. 10 ng/mL) and were incubated at 37 °C and 5% CO_2_ c. After 6–8 days, the cells strongly adhered to the plastic dish. The adherent differentiated cells were then scraped and assessed for macrophage marker (F4/80) before plating them for HEV infection.

### 2.6. Isolation of Human Monocytes and Differentiation into Macrophages In Vitro

Human peripheral blood was taken from the antecubital vein of healthy volunteers (n = 3), and the buffy coat was separated using Ficoll-Hypaque density gradient centrifugation. Human monocytes were obtained from human blood by negative selection of freshly isolated PBMCs using a Pan Monocyte Isolation Kit, human (Miltenyi Biotec, Paris, France), with a typical purity of 90–98% as assessed by flow cytometry after staining with PE anti-human CD14 (BioLegend, USA) and FITC anti-human CD16 antibodies (BioLegend, USA). Monocytes were cultured in RPMI media (Sigma Aldrich Chemie GmbhMunich, Germany) containing 1% penicillin/streptomycin, supplemented with 10% FBS (Gibco, ThermoFisher Scientific), and cultured at 37 °C and 5% CO_2_ in the presence Granulocyte-Macrophage Colony-Stimulating Factor (GM-CSF, conc. 10 ng/mL) to generate human macrophages (HMACs) within 6–8 days [28]. Following differentiation, HMACs adhered strongly to the plastic plate. Expression of the macrophage marker CD68 was tested by flow cytometry. Cells were fixed and permeabilized using Intracellular Fixation/Permeabilization concentrate (eBiosience, Thermofisher scientific, Grand Island, NY, USA) and were then stained with APC anti-human CD68 antibody (BioLegend, USA). For bone marrow, the cell suspension was dissolved in RBCs lysis buffer (1X, Thermofischer), filtered, and dissolved in RPMI media containing 1% penicillin/streptomycin and macrophage-colony stimulating factor (GM-CSF, conc. 10 ng/mL) as described in the previous sections. The differentiated macrophages were identified by the specific marker (CD68) and adherence property.

### 2.7. Infection of HMOs and HMACs with HEV Preparations

Infection of HMOs and HMACs with HEV inoculums was assessed as described previously [18]. Briefly, one day prior to infection, 2 × 10^4^ cells/well of HMOs and HMACs were seeded in 24-well plates. A filtered 10% w/v fecal preparation was added to the cells at a dose of 10^6^ IU/well and incubated for 6 h. Then, the inoculum was removed and replaced with fresh culture medium. Collections of supernatants were done at 0, 1, 3, 5, 7, 10, and 12-days post-infection. Supernatants from each time point were stored at −80 °C, and processing of all samples was done at the same time. As proof of concept for HEV replication, HMOs and HMACs were treated with RBV (Sigma–Aldrich, Munch, Germany) at final conc. 50 µM and challenged with HEV inoculum as described previously [18,29,30,31]. For infectivity assay, cell lysate was prepared from BMDM challenged with HEV-1 at day 15 pi as described previously [32]. Briefly, BMDMs were trypsinized at 37 °C for 3–5 min, and then the trypsin was neutralized using RPMI media containing 10% FBS. After the centrifugation step, the cell pellets were used to prepare the cell lysate by vigorous vortexing in the presence of a high salt concentration to release the intracellular viral particles. HMACs were seeded in a 96-well plate and challenged with HEV-1 cell lysate. The HEV RNA load and HEV Ag were assessed in the supernatant of the infected cells.

### 2.8. Quantification of HEV RNA by qRT-PCR

Quantification of HEV RNA was done as described previously [32,33,34,35]. Briefly, viral RNA was extracted from cell culture supernatants using the QIAamp Viral RNA Mini Kit (Qiagen, Hilden, Germany) according to the manufacturer’s instructions. HEV RNA was quantified using primers targeting HEV ORF2/3 as described previously [32,33,34]. The limit of quantification (LOQ) of our assay was 300 IU/mL for undiluted samples.

### 2.9. Detection of HEV ORF2 Ag and ds-RNA in the Infected Cells

Detection of HEV ORF2 Ag in the infected cells was done by flow cytometry as described previously [18]. Briefly, HMOs and HMACs were challenged with the stool-derived HEV-1 and/or HEV-3 as described; the cells were fixed at day 12 post infection and permeabilized using eBioscience™ Fixation/Permeabilization Concentrate (ThermoFischer Scientific, USA) according to the manufacturer’s instruction and then stained with antibody 1E6 clone (Millipore, Billerica, MA, United States; dilution 1/1000) that targets the HEV ORF2 protein. Goat anti-mouse IgG conjugated with Alexa488 (Invitrogen, Waltham, MA, USA) was used as a secondary antibody according to the manufacturer’s instructions. For detection of ds-RNA, the cells were fixed using cold methanol, permeabilized by 0.5% Triton X100, and stained with mouse monoclonal anti-dsRNA J2 Ab (Millipore; dilution 1/200). Goat anti-mouse IgG conjugated with Alexa647 (Invitrogen) was used as a secondary antibody (dilution 1/1000), and DAPI was used for nuclei staining.

### 2.10. Monitoring of the Extracellular HEV Capsid Protein by ELISA

Detection of HEV ORF2 Ag in cell culture supernatants was performed using the HEV-Ag ELISA^Plus^ assay (Wantai Biological Pharmaceutical Co., Bejing, China) according to the manufacturer’s instructions, with slight modifications in the procedure of the cut-off (C.O.) calculation as described previously [36].

### 2.11. Measurement of the Level of Inflammatory Cytokines Released after HEV Infection

Supernatants, collected from uninfected and HEV-infected HMACs at day 12 pi were tested for IL-12, IL-6, IL1-β, MCP-1, TNF-α, and IFN-γ cytokines by ELISA kits (R&D Systems, Minneapolis, MN, USA) according to the manufacturer’s instructions. The level of these cytokines was compared in HEV-infected cells versus the uninfected cells with the same cell number. As a control group, HMACs were challenged with Ultraviolet (UV)-inactivated HEV inoculums and assayed for the same cytokines. UV inactivation was performed in an UV transilluminator for 30 min

### 2.12. Statistics

Statistical analyses were performed using GraphPad Prism software 6 (GraphPad Software, La Jolla, CA., USA) using an unpaired *t*-test. *p* < 0.05 was considered significant.

## 3. Results

### 3.1. Murine Monocytes, Macrophages, and BMDMs are Not Susceptible to HEV Infection

**Figure 1:**
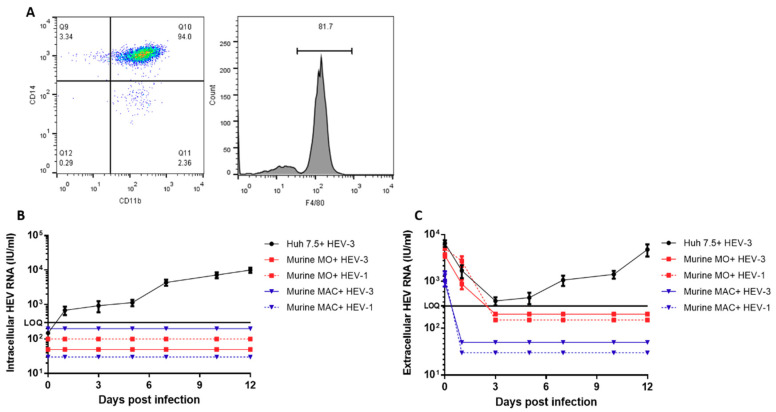
Characterization of murine monocytes and macrophages and infection with HEV inoculums (**A**) The isolated murine monocytes were analyzed by flow cytometry to check for purity, and isolated cells were stained with anti-CD14 and anti-CD11b (left). After differentiation into macrophages, the cells expressed a high level of F4/80 (right). Infection of murine monocytes (MMOs red) and murine macrophages (MMACs-blue) with HEV-1 (dotted line) and HEV-3 (solid line) inoculums. Intracellular HEV RNA (**B**) and extracellular HEV RNA (**C**) were quantified in the supernatants by qPCR. Huh7.5 (black) challenged with HEV-3 was used as a positive control. LOQ: limit of quantification. Depicted are the mean values of three independent experiments  ±  SEM.

Primary murine monocytes (MMOs) were isolated from the blood of Balb/c mice (n = 3) as described in the previous section. Analysis of the isolated cells by flow cytometry showed that the cells expressed the monocyte (MO) marker (CD14, C11b) (Figure 1A). MMOs were challenged with HEV-1 and HEV-3. The intracellular and extracellular HEV RNA load was under the limit of quantification (LOQ) for all tested time points (Figure 1B,C). Then, we aimed to determine whether primary murine macrophages (MMACs, positive F4/80 cells) are susceptible to HEV infection. MMOs were differentiated to MMACs, and then we challenged these cells with the HEV inoculums. Similar to MMOs, HEV did not replicate in F4/80-positive MMACs (Figure 1B,C). In both MMOs and MMACs, the ds-RNS, i.e., intermediate of the viral genome replication, was not detectable by IF (Figures S1 and S2). As a positive control, Huh7.5 cells were challenged with HEV-3, and the virus replicated efficiently in these cells as shown by the increase in the viral load over time and the detection of ds-RNA in the infected cells (Figure 1B,C, and Figure S1). Also, BMDMs isolated from the same mice (n = 3) and macrophage cell line (RAW 264.7 cells) were not susceptible to HEV infection 

### 3.2. Human-Derived Monocytes and Macrophages are Susceptible to HEV Infection

**Figure 2:**
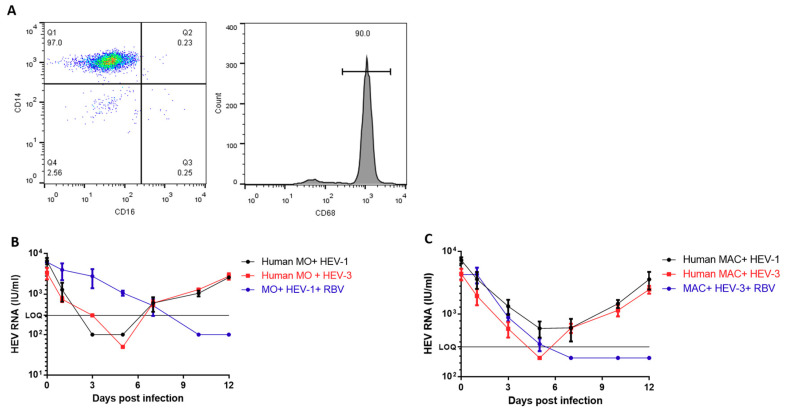
Characterization of human monocytes and macrophages and infection with HEV inoculums (**A**) The isolated human monocytes were analyzed by flow cytometry to check for purity, and isolated cells were stained with anti-CD14 and anti-CD16 (left). After differentiation into macrophages, the cells expressed high levels of the intracellular marker CD68 (right). (**B**) Infection of human monocytes (HMOs) with HEV-1 (black) and HEV-3 (red) inoculums. HMOs challenged with HEV-1 were treated with ribavirin (RBV-50 um) (blue). Extracellular HEV RNA was quantified in the supernatants by qPCR. (**C**) Infection of human macrophages (HMACs) with HEV-1 (black) and HEV-3 (red) inoculums. HMACs challenged with HEV-3 were treated with ribavirin (RBV-50 um) (blue). Extracellular HEV RNA was quantified in the supernatants by qPCR. LOQ: limit of quantification. Depicted are the mean values of three independent experiments  ±  SEM.

Primary human monocytes (HMOs) were isolated from the blood of healthy volunteers (n = 3) as described in the previous section. Analysis of the isolated cells showed that the cells expressed the MO marker (CD14+). Differentiation of HMOs into human macrophages (HMACs) was done in vitro, and the differentiated cells expressed the MAC marker (CD68) (Figure 2A); the HMACs were also recognized by their adherence property. HMOs and HMACs from the same donors were challenged with HEV-1 and HEV-3 inoculums. The HEV RNA started to increase in the supernatant of infected HMOs by day 7 post-infection (p.i) for HEV-1-and HEV-3-infected cells. The viral load increased over time, reaching 3.1 × 10^3^ IU/mL in the supernatant of infected cells by day 12 pi (Figure 2B). Similarly, the extracellular viral load started to increase in HMACs-infected cells at day 7 pi, reaching 4.8 × 10^3^ IU/mL by day 12 p.i in the supernatants of the infected cells (Figure 2C). Generally, we did not see a significant difference in the replication efficiency and viral load between HEV-1 and HEV-3 viruses on HMOs and HMACs. As proof of concept for HEV replication in these cells, we assessed the effect of RBV on HEV replication. RBV treatment (final concentration 50 µm) inhibited the replication of HEV-1 and HEV-3 in HMOs and HMACs as shown by the gradual decrease in HEV RNA titers in the supernatants of the treated cells until it decreased to below the LOQ (Figure 2A,B and Figure S2) Contrary to the primary HMOs and HMACs, HEV was not infectious to the monocytic leukemic cell line (THP-1), and the HEV load decreased over time until it became undetectable 

### 3.3. Detection of the HEV Capsid Protein and dsRNA in the Infected HMOs and HMACs

**Figure 3:**
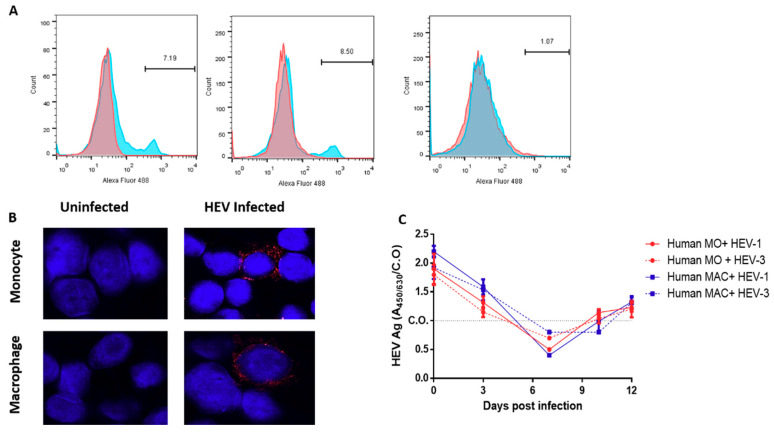
Detection of the HEV capsid protein and ds-RNA in HMOs and HMACs challenged with HEV inoculums (**A**) Representative gating strategy showing the expression of HEV ORF2 Ag in HMOs and HMACs infected with HEV. (left): HMOs infected with HEV-1. (Middle): HMACs infected with HEV-3, and (right): mock infected cells. Red histograms represent cells stained with the secondary A488 conjugated anti-mouse antibodies alone; blue histograms represent cells stained by mouse anti-HEV-ORF2 followed by A488-conjugated anti-mouse antibody. (**B**) Representatives showing HMOs (upper panel) or HMACs (lower panel) either uninfected (left side) or infected with HEV preparations (right side) were fixed and stained with anti-ds-RNA (J2 Ab) (red). DAPI was used for nuclear staining (blue) (Scale bars, 20 µm). Representative showing infection of HMOs with HEV-1 and infection of HMACs with HEV-3. (**C**) Supernatants collected from HEV-1 (solid line) and/or HEV-3 (dotted line)-infected HMOs (red) and HMACs (blue) were tested for HEV ORF2 Ag by ELISA, C.O is the cut off. Depicted are the mean values of three independent experiments  ± SEM.

To examine the expression of the HEV capsid protein in HMOs and HMACs, we assessed HEV ORF2 Ag expression in HEV-infected HMOs and HMACs on day 12 pi by flow cytometry. Uninfected HMOs and HMACs served as a negative control. We detected intracellular HEV ORF2 Ag inside the infected cells (Figure 3A); the percentage of HEV-ORF-2 positive cells was (7–10%) in the infected cells for both HEV-1 and HEV-3. Most positive-sense RNA viruses produce ds-RNA in infected cells as an intermediate of genomic RNA replication. Therefore, we assessed the presence of ds-RNA in HEV-infected HMOs and HMACs. We detected the ds-RNA in HMOs and HMACs (Figure 3B); the percentage of infected cells was 8 and 10% in HMOs and HMACs, respectively. To assess the HEV assembly and release from the infected HMOs and HMACs cells, the level of extracellular HEV ORF2 Ag was monitored in the supernatants of HEV-infected cells by ELISA. We found that the level of HEV ORF2 Ag started to increase by day 10 pi, and the Ag level (as determined by A/C.O) increased over time, indicating the assembly and release of HEV particles from the infected cells (Figure 3C). In parallel with the HEV RNA load, the level of HEV ORF2 Ag was not significantly different in the supernatant of HEV-1-and HEV-3-infected cells regardless of the challenged cells.

### 3.4. Replication of HEV in Human Bone Marrow-Derived Macrophages (BMDMs)

**Figure 4:**
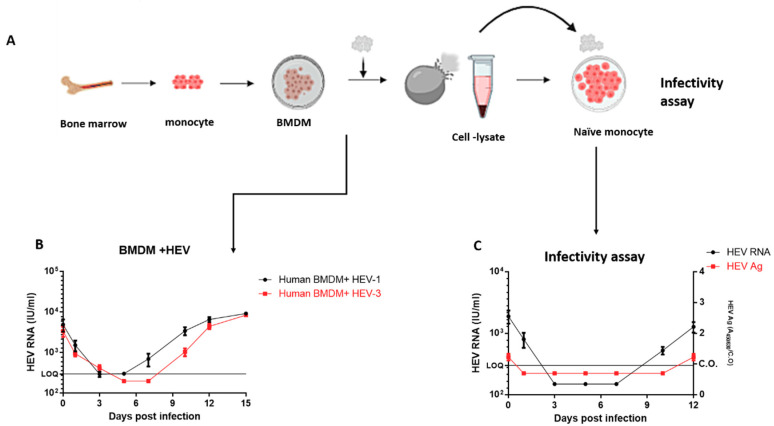
Infection of human BMDMs with HEV inoculums (**A**) Schematic flow showing differentiation of macrophages from bone marrow and preparation of cell lysate for infectivity assay (**B**) Infection of human BMDMs with HEV-1 (black) and HEV-3 (red) inoculums. Extracellular HEV RNA was quantified in the supernatants by qPCR. LOQ: limit of quantification. Depicted are the mean values of three independent experiments ± SEM. (**C**) HMOs were inoculated with cell lysate derived from BMDM on day 15 pi that contained HEV-1. HEV RNA (black; IU/mL, left *Y*-axis) and HEV ORF2 Ag (red; A_450/630_/C.O., right axis) were measured at different time points after infection. LOQ: limit of quantification. C.O.: cut-off.

Bone marrow was collected from healthy donors (n = 3), and the cells were differentiated in vitro into macrophages (BMDMs) (Figure 4A). We challenged BMDMs with HEV-1 and HEV-3 inoculums. In parallel with the previous data, extracellular HEV RNA was detected on day 7 and day 10 pi in HEV-1- and HEV-3-infected BMDM, respectively. The viral load increased over time and reached 1 × 10^4^ IU/mL by day 15 pi (Figure 4B). The viral load in the supernatant of BMDM was slightly higher than the viral load in the supernatant of HMOs and HMACs regardless of the virus genotype. Similar to our previous observation, we did not notice a significant difference in the replication efficiency of HEV-1 and HEV-3 with respect to BMDMs. To assess the infectivity of the HEV particles released from BMDMs, cell-lysate-derived HEV-1, prepared from infected BMDMs on day 15, was used as inoculum to infect naive HMOs (Figure 4A). HEV RNA was detectable in these supernatants of infected cells, and the viral loads increased with time. In addition, HEV Ag increased extracellularly, suggesting that the released HEV particles from BMDMs were infectious (Figure 4C).

### 3.5. The Level of Proinflammatory Cytokines Was Elevated in the Supernatant of HEV-Infected HMACs

**Figure 5:**
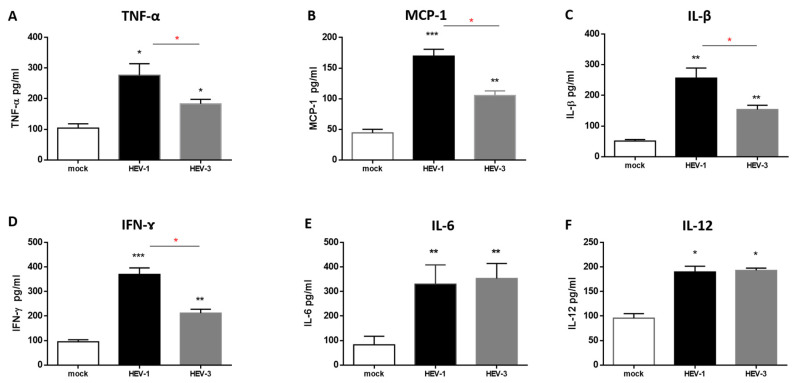
Immune response generated by HMACs after infection with HEV inoculums. Supernatants, collected from uninfected and HEV-infected HMACs on day 12 pi, were tested for TNF-α (**A**), MCP-1 (**B**), IL1-β (**C**), IFN-γ (**D**), IL-6 (**E**), and IL-12 (**F**) by ELISA. Depicted are the mean values of 3–5 independent experiments  ± SEM. * indicates *p* < 0.05, ** indicates *p* < 0.01, *** indicates *p* < 0.001 as determined by unpaired two-tailed Student’s t tests. * black compared HEV infected vs. uninfected. * red compared HEV-1-infected HMAC vs. HEV-3-infected HMACs.

Then, we wanted to assess the level of proinflammatory cytokines released due to HEV infection. Supernatants from HMACs challenged with HEV-1 and HEV-3 were collected on day 12 pi, and the levels of IL1-β, MCP-1, TNF-α, IFN-γ, IL-6, and IL-12 cytokines were measured by ELISA. We found that the level of the proinflammatory cytokines were higher in HEV-infected HMACs than in uninfected ones. The levels of IL1-β, MCP-1, TNF-α, and IFN-γ were significantly higher in HEV-1-infected HMACs than in HEV-3-infected HMACs. There was no statistical difference in the level of IL-12 and IL-6 between HEV-1- and HEV-3-infected cells (Figure 5). As a control group, HMACs were challenged with UV-inactivated HEV-1 and HEV-3, and we did not record a significant upregulation of the inflammatory cytokines produced (Figure S3).

## 4. Discussion

HEV causes acute, chronic, and extrahepatic manifestations. Chronic HEV infection develops in immunocompromised patients such as HIV-infected patients, leukemia patients, and patients with organ transplants [2]. The etiology and source of chronic HEV infection are not completely understood. Our results raise the possibility that ongoing viral replication in PBMCs could be a source for infection and/or recurrence of the infection and development of chronicity, especially in immunocompromised patients. Likewise, other hepatotropic viruses such as HBV and HCV replicate in PBMCs which play a role in the pathogenesis of chronic infections, infection of naïve allograft organs following liver transplantation, and/or in reactivation of these infections in immunocompromised patients [20]. Extrahepatic manifestations have been reported in association with HEV infection such as neurological disorders, glomerulonephritis, and hematological disorders [16,17]. HEV could enter the extrahepatic targets through circulation via PBMCs. In addition, the replication of HEV in HMOs, HMACs, and BMDMs could be linked directly or indirectly with hematological manifestations associated with HEV infection such as hepatitis-associated aplastic anemia, pure red cell aplasia, secondary hemophagocytic syndrome, monoclonal gammopathy of undetermined significance, severe thrombocytopenia, autoimmune hemolytic anemia, and hemolytic anemia [24]. Similarly, Wünschmann et al. reported that HAV replicated in HMOs and diminished their differentiation into HMACs, which could eventually lead to suppression of the myelomonocytic lineage and affect hematopoiesis [19]. Similarly, HCV infection can induce autoimmune hemolytic anemia, leukopenia, thrombocytopenia, and anemia of chronic disease, probably due to extrahepatic replication of HCV in bone marrow [22,37]. Moreover, transmission of HEV by whole blood and/or transfusion of blood products has been documented [7,38,39]. Blood products that were documented as sources of transfusion-transmitted (TT) HEV infection were plasma, red blood cells, apheresis platelets concentrate, cryoprecipitate, pooled granulocytes, and whole blood pooled platelet concentrates [38,39]. TT HEV can evolve to chronicity, especially in immunosuppressed patients [39]. The replication of HEV in PBMCs could be a potential source of infection for other blood cells and/or products and hence a cause of TT HEV infection.

In this study, we assessed the replication of HEV in murine and human myeloid cells. Although mice are considered a query model for HEV [40,41], we tested the susceptibility of MMOs, MMACs, and murine BMDMs to HEV. We previously reported that non-transplanted immunocompromised mice (uPA^+/+^/SCID and FRG) were not susceptible to HEV-1 and HEV-3 infection [32,33,36]. Using an elegant human liver chimeric mice, we reported that HEV-1 and HEV-3 replicated in the human hepatocytes embedded inside the murine liver, but not in the kidney or brain of these mice which were murine in origin [32,33,34]. However, we detected HEV RNA in the spleen of HEV-infected humanized mice, but not in the spleen of a challenged non-transplanted mouse. We explained the former finding is probably due to the presence of viremia in humanized mice which was absent in non-humanized mice. However, we aimed to determine whether HEV particles circulated extracellularly in the mouse blood and/or whether HEV particles replicated inside the MMOs circulating in the blood and entered the spleen through splanchnic circulation. Therefore, we assessed the susceptibility of the primary murine-derived mononuclear cells (monocytes, macrophages, and BMDM) and murine myeloid cell line (RAW 264.7 cells) to HEV infection. HEV RNA was not detectable in the cells previously challenged with HEV inoculums, suggesting that murine myeloid cells are resistant to HEV infections. This finding is not surprising to us, since murine liver, the main target for HEV replication, is also resistant to infection by these isolates [32,33,34,36].

Then, we determined whether human PBMCs are permissive to HEV Infection. We infected primary HMOs and HMACs with HEV inoculums and then we assessed HEV markers in the challenged cells. We found that HEV RNA and HEV ORF2 Ag increased over time in HMOs and HMACs challenged with HEV inoculums. Similar findings were recorded using human BMDMs challenged with HEV. In addition, the HEV capsid protein was detected intracellularly alongside dsRNA which is an intermediate of viral genome replication. Several studies used dsRNA as a marker of viral replication, especially positive-sense RNA viruses such as HCV [42], the Sindbis virus [43], rubella virus [44], Coronavirus [45], dengue virus [46], Picornavirus [47], etc. In addition, dsRNA was detected in the liver biopsy of a patient diagnosed with acute cholestatic HEV infection, suggesting that ds RNA could be a marker of HEV replication [48]. Collectively, our data suggested that HEV replicates inside HMOs, HMACS, and BMDMs, and the virus could complete the life cycle in these cells. Therefore, these cells represent another target for extrahepatic HEV replication. Several studies reported other targets that support HEV replication and represent sites for extrahepatic HEV replication such as neuron, placenta, and endometrial stromal cells [18,29,31].

In this study, we did not record a significant difference in the replication of HEV-1 and HEV-3 in HMOs, HMACs, and BMDMs. Although HEV-1 is more virulent than HEV-3 in infected patients and in vivo animal model [3,41,49], HEV-3 replicates more efficiently in the hepatoma-derived cell line, neuron-derived cell line, and placenta-derived cell line in vitro [18,29,31]. Some HEV-3 strains were adapted to grow in cell culture due to a recombination event resulting in an insertion of 174 ribonucleotides (58 amino acids) of the human ribosomal protein S17 gene into the viral genome [50]. While the replication of HEV-1 is inhibited in vitro at multiple levels [51]. On the other hand, HEV-1 replicates more efficiently in primary cells derived from maternal decidua, fetal placenta, the placental stromal cells, and endometrial stromal cell [18,30]. The latter finding is because HEV-1 causes most of the complications associated with HEV infection during pregnancy, while the course of HEV-3 during pregnancy is mild to moderate [17,52]. The replication of HEV-1 and HEV-3 in PBMCs in vivo has not yet been reported. Therefore, the clinical outcomes resulting from the replication of each genotype in PBMCs are not known. Future studies need to ascertain this point.

In this study, HEV could not replicate in the human monocyte cell line (THP1), which originated from a patient with acute leukemia. While HEV replicates in primary HMOs and HMACs. HEV-1 replicates efficiently in primary human hepatocytes, while its replication in the hepatoma-derived cell line is reduced or inhibited [18,53,54]. The primary cells for HEV replication are advantageous over cancer cell lines for being more physiologically relevant, with higher cell polarity and susceptibility to pan-genotypes [55]. However, we could not exclude the possibility of HEV replication in myeloid cell lines. It is likely that the use of different HEV isolates/strains, infection with higher viral inoculum, and/or other methodology such as transfection could prove the replication of HEV in this cell line

Our results suggest that HEV replicates inside HMOs and HMACs and these cells could be a reservoir and a source of HEV infection for other organs. Although the viral load released in the culture supernatant was low, the presence of dsRNA and the detection of intracellular and extracellular HEV capsid proteins confirmd the complete replication of HEV in these cells. In a swine model, both positive (+)-strand HEV RNA and negative (−)-strand HEV RNA were detected in the lymph node of pigs inoculated with swine-derived HEV-3 and human-derived HEV-3, suggesting that a lymph node is a site for extrahepatic HEV replication [56]. Similarly, both (+) and (−)-strand HEV RNA were detected in the lymph node and to a lesser extent in the tonsils of rabbits infected with swine-derived HEV-4 [57]. Lymph node contains immune cells such as T-lymphocytes, B-lymphocytes, plasma cells, and macrophages. Based on our current knowledge, there is no report on the replication of HEV in T and B lymphocytes, and it is possible that HEV replicated in macrophages residing in the lymph nodes. On the other hand, Ippagunta et al. detected (+)-strand HEV RNA in the PBMCs of acute HEV-infected patients, but they could not detect (–)-strand RNA using a strand-specific rTth assay [25]. However, the authors mentioned that the absence of (−)-strand HEV RNA may not exclude the replication of HEV in PBMCs, especially in immunocompromised patients. Moreover, the authors claimed that the relatively lower sensitivity of the strand-specific rTth RT-PCR assay was one of the limitations of their study, and a more sensitive technique is required to confirm their findings [25]. Herein, we used dsRNA as a marker for HEV intermediate genome replication in PBMCs which could be more sensitive and reliable than strand-specific RT-PCR assay.

In this study, we found the levels of proinflammatory cytokines such as MCP-1, IL-6, TNF-α, IL-β, IFN-γ, and IL-12 were higher in HEV-infected HMACs than in control cells. IL-12 and IFN-γ also play a role in the activation of adaptive immune responses, especially T-helper 1. Similarly, Saravanabalaji et al. isolated PBMCs from acute HEV patients, fulminant hepatitis failure (FHF) patients, and controls, and they stimulated these PBMCs with the recombinant ORF2 peptide (rORF2p) of HEV-1. They found that IL-12 and IFN-γ were higher in acute HEV patients than in controls, while TNF-α and IL-2 levels were similar in both groups [58]. IFN-γ, IL-2, and TNF- α were significantly higher in patients with FHF than in acute HEV patients [58]. Kumar et al. reported that significantly higher levels of TNF-α, IL-6, IFN-γ, and TGF-β1 were present in HEV-infected pregnant women compared to non-pregnant women and controls, and these four cytokines determined the pregnancy outcome [59]. Also, Taherkhani et al. reported that PBMCs, isolated from recovered HEV patients, produced significantly higher amounts of IFN- γ and IL-12, but not IL-10 and IL-4 compared with control groups upon stimulation with truncated HEV ORF2 of HEV-1 in vitro [60]. Likewise, Srivastava et al. reported that IFN-γ levels in the supernatants and IFN-γ mRNA transcripts in cells were elevated in ORF2-stimulated PBMCs in acute HEV patients, while the levels of IL-2 or TNF-*α* were unchanged. They also reported that the serum IL-1*β* level was higher in acute HEV patients than in the controls [61]. Collectively, our results agree with the previous reports. Importantly, based on our current knowledge, this study is the first report that shows the immune response of PBMCs against whole HEV particles; most of the previous studies focused on the stimulation of PBMCs with truncated or partial HEV ORF2. Interestingly, we found that the levels of MCP-1, TNF-α, IL1-β, and IFN-γ were higher in the supernatant of HEV-1-infected PMCS than in HEV-3-infected cells, although the viral load was comparable in both groups. HEV-1 is more virulent than HEV-3 and it causes acute infection with more severe outcomes in certain situations, for example, pregnancy [3]. Likewise, HEV-1 induces more immune responses in humanized mice than HEV-3 [32,62].

## 5. Conclusions

Our study is the first report that shows evidence of HEV replication in PBMCs in vitro. The fact that HEV persists and multiplies in HMOs, HMACs, and BMDMs is an important finding in understanding HEV pathogenesis. PBMCs could be a reservoir for HEV and the source of chronic, recurrent infection, as well as extrahepatic manifestations. HEV infection of BMDMs could be related to hematological disorders associated with HEV infection.

## Supplementary Materials

The following are available online at https://www.mdpi.com/2076-393X/8/2/239/s1, Figure S1: Assessment of ds-RNA in the murine monocytes, macrophages challenged with HEV inoculums. Infection of murine macrophages (MMACs) with HEV-1 (A) and HEV-3 (B) inoculums. Ds-RNA was assessed in the challenged cells by IF. (C) Huh7.5 challenged with HEV-3 was used as a positive control, Figure S2: Test the effect of RBV on HEV replication on HMOs and HMACs. Infection of human monocytes (HMOs) with HEV-3 (A), and human macrophages (HMACs) with HEV-3 (B). These cells were treated with ribavirin (RBV-50uM). Extracellular HEV RNA was quantified in the supernatants by qPCR. LOQ: limit of quantification. Depicted are the mean values of three independent experiments ± SEM, Figure S3: Test the effect of UV-inactivated HEV inoculums on the release of inflammatory cytokines from HMACs. Supernatants, collected from uninfected, UV-inactivated HEV-1, UV-inactivated HEV-3 HMACs at day. 12 pi were tested for TNF-α (A), MCP-1 (B), IL1-β (C), IFN-ɤ (D), IL-6 (E), and IL-12 (F) by ELISA. Depicted are the mean values of 3-5 independent experiments ± SEM.
