# Supplementary material for: Replication of Hepatitis E Virus (HEV) in Primary Human-Derived Monocytes and Macrophages In Vitro
Source: Vaccines (Basel). 2020 May 21;8(2):239. doi: 10.3390/vaccines8020239 (PMC7349946; doi:10.3390/vaccines8020239)
Supplement: Supplementary file 1 [file vaccines-08-00239-s001.pdf]

Supplementary data

1- Assessment of ds-RNA in the murine monocytes, macrophages challenged with HEV inoculums

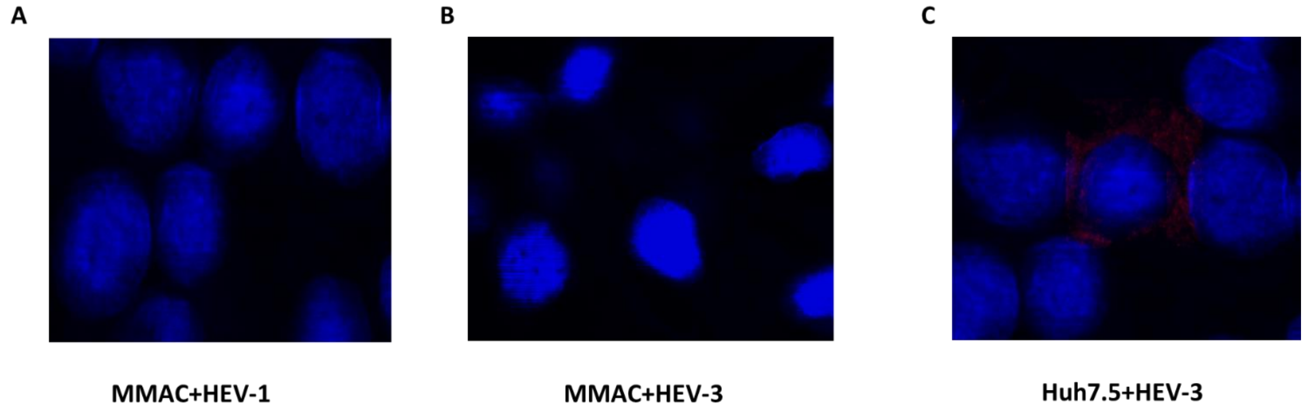

Fig S1: Assessment of ds-RNA in the murine monocytes, macrophages challenged with HEV inoculums

Infection of murine macrophages (MMACs) with HEV-1 (A) and HEV-3 (B) inoculums. Ds-RNA was assessed in the challenged cells by IF. (C) Huh7.5 challenged with HEV-3 was used as a positive control.

2- Test the effect of RBV on HEV replication on HMOs and HMACs

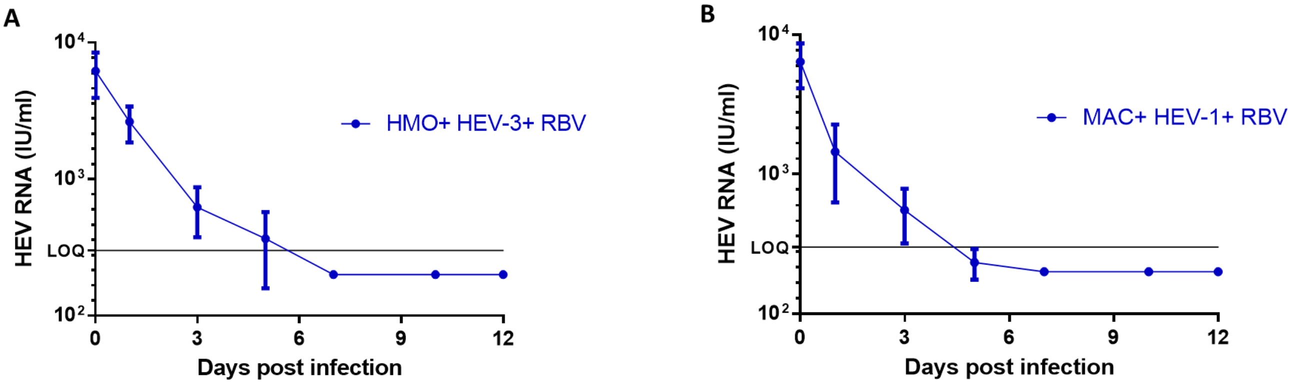

Fig S2: *Test the effect of RBV on HEV replication on HMOs and HMACs*

Infection of human monocytes (HMOs) with HEV-3 (A), and human macrophages (HMACs) with HEV-3 (B). These cells were treated with ribavirin (RBV-50uM). Extracellular HEV RNA was quantified in the supernatants by qPCR. LOQ: limit of quantification. Depicted are the mean values of three independent experiments ± SEM.

3- *Test the effect of UV-inactivated HEV inoculums on the release of inflammatory cytokines from HMACs*

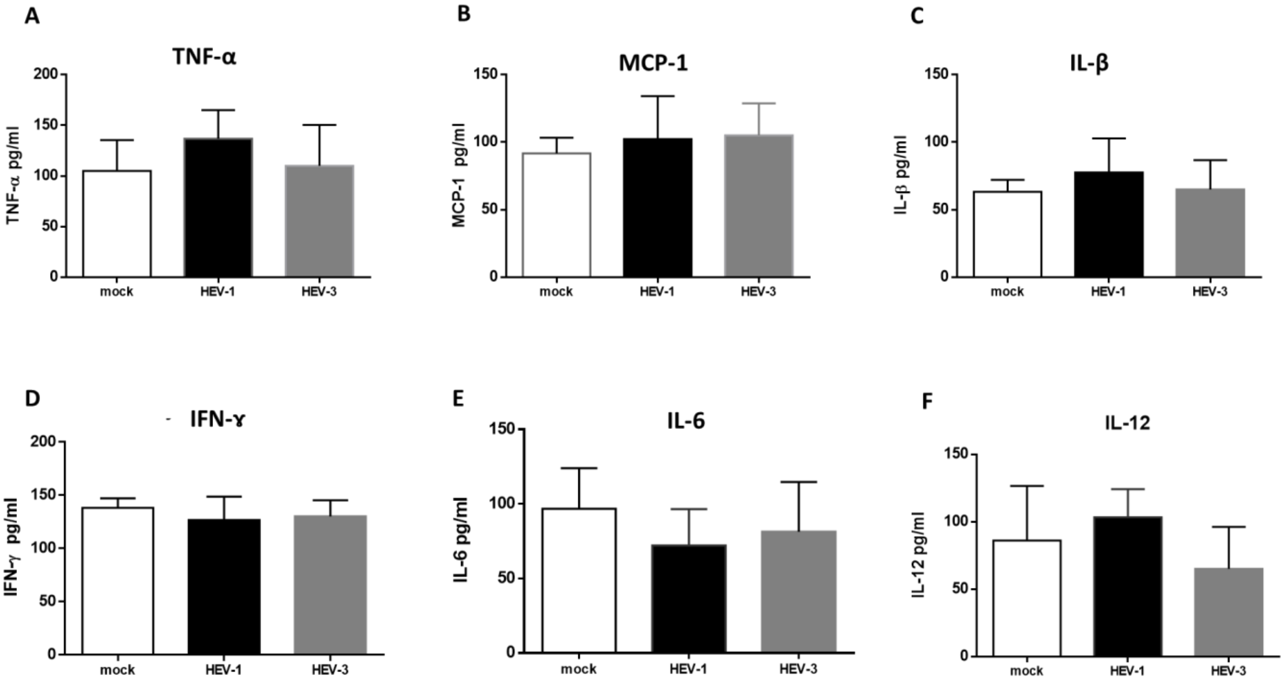

Fig S3 *Test the effect of UV-inactivated HEV inoculums on the release of inflammatory cytokines from HMACs*

Supernatants, collected from uninfected, UV-inactivated HEV-1, UV-inactivated HEV-3 HMACs at day 12 pi were tested for TNF-α (A), MCP-1 (B), IL1-β (C), IFN-ɤ (D), IL-6 (E), and IL-12 (F) by ELISA. Depicted are the mean values of 3-5 independent experiments ± SEM.
